# Claudin-17 Deficiency in Mice Results in Kidney Injury Due to Electrolyte Imbalance and Oxidative Stress

**DOI:** 10.3390/cells11111782

**Published:** 2022-05-29

**Authors:** Mir S. Adil, Varun Parvathagiri, Arti Verma, Fang Liu, Madhuri Rudraraju, S. Priya Narayanan, Payaningal R. Somanath

**Affiliations:** 1 Clinical and Experimental Therapeutics, College of Pharmacy, University of Georgia, Augusta, GA 30912, USA; miradil@stanford.edu (M.S.A.); vparvathagiri@augusta.edu (V.P.); averma2@augusta.edu (A.V.); fliu1@augusta.edu (F.L.); mrudraraju@augusta.edu (M.R.); pnarayanan@augusta.edu (S.P.N.); 2 Research Division, Charlie Norwood VA Medical Center, Augusta, GA 30912, USA

**Keywords:** *CLDN17*, kidney injury, plasma electrolytes, reactive oxygen species, inflammation

## Abstract

The multi-gene claudin (*CLDN*) family of tight junction proteins have isoform-specific roles in blood–tissue barrier regulation. *CLDN17*, a putative anion pore-forming *CLDN* based on its structural characterization, is assumed to regulate anion balance across the blood-tissue barriers. However, our knowledge about *CLDN17* in physiology and pathology is limited. The current study investigated how *Cldn17* deficiency in mice affects blood electrolytes and kidney structure. *Cldn17^−/−^* mice revealed no breeding abnormalities, but the newborn pups exhibited delayed growth. Adult *Cldn17^−/−^* mice displayed electrolyte imbalance, oxidative stress, and injury to the kidneys. Ingenuity pathway analysis followed by RNA-sequencing revealed hyperactivation of signaling pathways and downregulation of SOD1 expression in kidneys associated with inflammation and reactive oxygen species generation, demonstrating the importance of *Cldn17* in the maintenance of electrolytes and reactive oxygen species across the blood-tissue barrier.

## 1. Introduction

Claudins (*CLDNs*) are critical tight junction (TJ) proteins involved in the transcellular and paracellular transport coupling [1,2], ion and electrolyte homeostasis, and blood-tissue-barrier maintenance [3,4]. Besides ion selectivity, specific *CLDNs* are also responsible for cell-cell barrier formation and regulation of vascular permeability [5]. Blood–tissue barriers safeguard organs and control vital physiological processes through highly co-ordinated paracellular and transcellular transport [6]. Whereas injury to the alveolar-capillary unit causes pulmonary edema [7], a similar condition in the kidneys causes renal arterial stenosis [8]. Modulations in *CLDN* expression in the blood-tissue barrier often result in inflammation [9] and the generation of reactive oxygen species (ROS), and vice versa [10,11]. As a prime example, *CLDN4* was upregulated via an ROS-mediated pathway in Madin-Darby canine kidney (MDCK) cells in hyperosmolar conditions [12]. Likewise, other pore-forming *CLDNs* could also play a role in ROS homeostasis.

A major pathway known to regulate the expression of *CLDNs* in vascular endothelial cells (ECs) is the Akt signaling pathway [13,14,15,16,17]. The over-expression of VE-cadherin in the adherens junctions (AJs) resulted in increased *CLDN5* expression in ECs via activation of Akt, in turn, blocking the suppressive effects of FoxO (Forkhead Box-O)-1/3a and β-catenin [13]. Similarly, treatment with angiopoietin-1 activated Akt to promote *CLDN5* expression in human microvascular ECs [14]. Although vascular endothelial growth factor (VEGF), a short-term vascular permeability inducer, promoted its effects via Src activation, it protected the EC-barrier in the long-term via activation of Akt and suppression of FoxO and β-catenin to promote *CLDN5* expression [14,15,17]. Our past studies have revealed that Akt1-deficient ECs express significantly reduced levels of several *CLDNs* such as *CLDN3-7, CLDN9, CLDN17,* and *CLDN19*, with the highest decrease observed in *CLDN17* [14], suggesting that these *CLDNs* may likely be primary mediators of blood-tissue barrier regulation.

*CLDN17* is widely expressed in the brain and the kidneys [18] and forms a distinct anion-selective channel, which is structurally and functionally different from the paracellular cation channels [19]. Interestingly, it belongs to a class of ‘intron-less genes’ that constitutes ~3% of total human genes [20] that regulate the evolution of tissue-specific functions in mice and have diverse functions [21]. In the kidneys, *CLDN17* is predominantly expressed in the proximal segments of the nephron [22]. A recent study reported the downregulation of *CLDN17* associated with increased *CLDN3* and *CLDN8* in bronchial epithelial cells (EpCs) on dexamethasone-induced glucocorticoid activation [23]. It has also been reported that *CLDN17* replaces *CLDN8* to recruit occludin *(OCLN)* in human bronchial EpCs [23], suggesting that *CLDN17* assumes the role of *CLDN8* in tissues where *CLDN8* is absent and *vice versa*, e.g., *CLDN8* is abundant in the airways but not in the alveolar EpCs [23], suggesting that *CLDN17* may be a primary mediator of the blood-tissue barrier in various organs, including kidneys [24].

As of today, the precise role of *CLDN17* in physiology and pathology is unknown, except for some cancer cells in vitro [25,26,27]. Literature on *CLDN17* is scarce, and the reagents and tools to study the role of *CLDN17* in cell–cell and blood–tissue-barrier regulation are limited. Although a few *CLDNs* have been studied in vivo employing knockout mouse models, no laboratory, to date, has studied the effects of *CLDN17* in a living organism. Hence, in the current study, we generated a *Cldn17^−/−^* mouse model on a C57BL/6 background using the CRISPR/Cas9 technology and characterized its phenotype to reveal the significance of the gene concerning electrolyte homeostasis, redox signaling, and inflammation, leading to kidney injury. Our study revealed an important role for *Cldn17* in kidney function via the regulation of serum electrolytes and tissue reactive oxygen species levels.

## 2. Materials and Methods

### 2.1. Generation and Maintenance of Cldn17^−/−^ Mice

All the mouse-related experiments were performed with the approval of Charlie Norwood Veterans Affairs Medical Center Institutional Animal Care and Use Committee (approval references #19-04-114 and #20-01-118) and followed the ARRIVE guidelines for reporting experiments involving mice. Ketamine and xylazine were administered intraperitoneally in combination for inducing anesthesia. Carbon dioxide asphyxiation followed by cervical dislocation was used for euthanizing undesired mice. To generate knockout mice (Horizon Discovery, Lafayette, LA, USA), ten suitable sgRNAs for CRISPR/Cas9 knockout in C57BL/6 mice zygotes were performed, and the most potent sgRNA with minimal off-target potential was assembled into the ribonucleoprotein complex with Cas9 endonuclease (gRNA Binding/PAM Site 5′→ 3′: TCGGTTTGGTTGGGACGATTGGG; Forward Primer 5′→ 3′: AGAAGACCAGGCACTCCTCT and Reverse Primer 5′→ 3′: TCATGCCGCAGATGCCAATA for genotyping). These were delivered into zygotes from C57BL/6 mice followed by embryo transfer into pseudo-pregnant females. Genomic PCR and DNA sequencing analyzed viable progenies for the presence of the desired mutation. Each small guide RNA was validated by transfection into cultured cells and assessed cutting efficiency by the Surveyor Cel 1 Mutation Detection assay. The two smaller cleavage bands added up to the size of the parental band. The ratio of the intensity of the cleavage bands relative to the parental band was used to assess cutting efficiency. Up to two selected transgenic founder mice were bred with C57BL/6 mice to produce F1 progeny heterozygous for the Cldn17^−/−^, as confirmed by the PCR-mediated genotyping and DNA sequencing analysis. Off-target analysis and on-target sequence confirmation in F1 mice were also performed, which consisted of PCR and sequencing of the top 10 predicted off-target sites. Cldn17^+/–^ mice were eventually bred to obtain homozygous Cldn17^−/−^ mice used in the study. A detailed genotyping protocol is provided in Supplementary Figure S7. Due to the size and growth-state differences in the young mice, all experiments were performed on 8–12-week-old mice.

### 2.2. RNA Isolation, cDNA Preparation, and qRT-PCR

Mice lungs were collected, RNA was extracted using an RNA isolation kit (RNeasy Plus, Qiagen, Valencia, CA, USA), and RNA quality was confirmed using a Nanodrop 2000 spectrophotometer (Thermo Scientific, Waltham, MA, USA). Complementary DNA (cDNA) was synthesized from 700 ng of RNA using RT2 First Strand kit (Qiagen) using a StepOne Plus thermal cycler and detection software (Applied Biosystems, Foster City, CA, USA), and quantitative real-time PCR (qRT-PCR) was performed using the RT2 SYBR Green ROX qPCR Mastermix (Qiagen, Hilden, Germany) in real-time PCR equipment (Applied Biosystems, Troy, NY, USA). Sample cDNA was amplified and quantified over many shorter cycles under the following conditions: an initial 10 min 95 °C period followed by 45 cycles of 95 °C for 15 s, 60 °C for 1 min, and 72 °C for 15 s. The threshold cycle (Ct) was determined using the exponential growth phase and the baseline signal from fluorescence vs. cycle number plots. The mouse primers used for the messenger RNA (mRNA) analysis of *Cldn17* were 5′-tctccctccggtactggaag-3’ (forward) and 5′-gctcctccaagttctcgc tt-3’ (reverse) and the primers for β-actin, used as a house-keeping control gene, were 5′-tttgagaccttcaacacccc -3’ (forward) and 5′-atagctcttctccagggagg -3’ (reverse). The list of primers used for the expression analysis of *Cldn* isoforms [28] in the mouse kidneys is listed in Supplementary Table S3.

### 2.3. Western Blot Analysis

It was performed as described in previously published studies [29,30]. Western analysis was performed on the cell lysates prepared using 1X RIPA lysis buffer (Millipore, Temecula, CA, USA) supplemented with protease and phosphatase inhibitor tablets (Roche Applied Science, Indianapolis, IN, USA). The DC protein assay reagent was used for protein estimation in the lysates (Bio-Rad Laboratories, Hercules, CA, USA). Approximately 30–40 μg of heat-denatured cell lysates prepared in Laemmli buffer were loaded onto the SDS-PAGE gels. Densitometry was performed using the NIH ImageJ software. Antibodies used include *Cldn17* from Abcam, Waltham, MA, USA (ab23333) and LSBio, Seattle, WA (LS-C3063), SOD1 (2770S, Cell Signaling Technology, Danvers, MA, USA), and β-actin (A5441, Sigma, St. Louis, MO, USA).

### 2.4. Analysis of Serum Electrolytes and Urine Parameters

WT and *Cldn17^−/−^* mice were subjected to retro-orbital puncture to collect around 800 to 1000 µL of blood. A part of the collected volume was used for a complete blood count. The rest was processed for the analysis of serum electrolytes and protein levels. The ion-specific electrode (ISE) method was used to assess serum sodium, potassium, and chloride levels. Urine was collected from WT and *Cldn17^−/−^* mice using metabolic cages to determine various parameters, including pH, EpCs, blood cells, and others.

### 2.5. Kidney Injury and Wet/Dry Ratio Analysis

Age- and gender-matched WT and *Cldn17^−/−^* mice were euthanized to collect their kidneys, then processed for histological examination and determination of tissue edema. Kidneys were fixed in 4% paraformaldehyde, and 10 μm thick sections were processed for H&E staining. Histological assessment of these slides was performed by examination of tissue fields by blinded reviewers based on scoring from 1 to 5, with one being the best and five being the worst (severe most injury), and averages of these scores were considered for statistical analysis. Mineralization, vacuolar degeneration, and Bowman’s capsule spacing were taken into consideration for kidney scoring. The kidneys and fecal pellets were weighed when fresh, followed by incubation in an oven for 72 h on sterile papers at 80 °C to remove all moisture content. The dry weights were then recorded to calculate the wet/dry ratios, which determine edema or fluid content. The fecal water content (%) was determined using the formula, (Average wet weight − verage dry weight) × 100/(Average wet weight + Average dry weight).

### 2.6. Genomics Data Co-Expression Analyses

Gene expression omnibus (GEO), a public functional genomics data repository (https://www.ncbi.nlm.nih.gov/gds last accessed on 15 May 2022), was used to study the co-expression regulation of *CLDN17* with other potential genes (including all CLDNs) that were identified by a separate literature search to play a substantial role in association with our gene of interest, which is *CLDN17*. A series of GEO datasets (GDS5453, GDS3510, GDS3941, GDS3568, GDS2750, GDS3489, GDS5383, GDS4302, GDS3177, GDS5040, GDS5279) were analyzed for expression of *CLDN17* and the identified genes. The trend in expression change upon intervention was recorded as high (upregulated), low (downregulated), or mixed (both upregulated and downregulated) irrespective of the statistical significance. These trends of the identified genes were then compared with the expression change in CLDN17 to record the extent of the similarity.

### 2.7. RNA-Seq and Bioinformatics Analysis

Kidneys were isolated from WT and *Cldn17^−/−^* mice and stored in Trizol^®^ before freeze-drying. Samples were then subjected to RNA isolation, and total RNA purity and concentration were evaluated by spectrophotometry using NanoDrop ND-1000 (Fisher Scientific, Hampton, NH, USA). Total RNA quality was assessed using the Agilent 2100 bioanalyzer (Agilent Technologies, Santa Clara, CA, USA) and assured of an RNA Integrity Number (RIN) greater than 5. Total RNA samples were processed for cDNA library preparations using TruSeq Stranded Total RNA kit (Illumina, San Diego, CA, USA), which depletes cytoplasmic ribosomal RNA. Briefly, 800 ng of total RNA was treated with an rRNA Removal Mix to deplete rRNA. Following purification, the RNA was fragmented into small segments, 200–300 bp in size, and converted to cDNA fragments. These cDNA fragments then had the addition of a single ‘A’ base and subsequent ligation of the adapter. The products were purified and enriched with PCR to create the final cDNA library. The prepared library was examined by a bioanalyzer and Qubit (Fisher Scientific, Hampton, NH, USA) to test library quality and quantity, respectively. The libraries were pooled and run on the NextSeq500 sequencing system using a 75-cycle paired-end protocol. BCL files generated by the NextSeq500 were converted to FASTQ files for downstream analysis. Reads which passed the quality control were aligned to the reference genome, starting with the STAR aligner. The generated BAM files in a comparative setup were imported to the following Cufflinks and Cuffdiff tools from Tuxedo Suite for outputting differentially expressed genes with log2-fold changes and q values having replicates. Genes with p-values of less than 0.05 were taken into consideration for bioinformatics analysis which was performed using gene set enrichment analysis (GSEA) and gene ontology (GO). R programming was utilized to generate graphs, including the volcano plot for the modulated genes, and the Panther database was used to identify signaling pathways based on significantly altered genes.

### 2.8. Ingenuity Pathway Analysis (IPA)

IPA database (Qiagen Bioinformatics, Toronto, Canada) transforms a list of genes into a set of relevant networks associated with pathology based on extensive records maintained in the Ingenuity Pathways Knowledge Base [31]. Highly interconnected networks are predicted to represent a significant biological function. Only those genes that were directly affected by the pathway of interest and *Cldn17* are shown.

### 2.9. ROS Analysis

Production of ROS was estimated in WT and *Cldn17^−/−^* mice with dihydroethidium (DHE) staining on kidney sections frozen using optimal cutting temperature compound as described in a previous study [32]. Stained images were then captured on confocal microscopy and subjected to fluorescence quantification using ImageJ software.

### 2.10. Analysis of Growth Hormone Expression

Growth hormone level was determined in the WT and *Cldn17^−/−^* mice using an ELISA kit from Novus Biologicals, Littleton, CO (NBP3-08143) according to the instructions provided by the manufacturer. Liver samples in PBS were used for the detection of growth hormone from pups and adult mice.

### 2.11. Statistical Analysis

Data are presented as mean ± s.d. or mean ± s.e.m. The ‘n’ value for each figure implies the number of samples in each group which was determined by power analysis. All the data were analyzed by parametric testing using the Student’s unpaired *t*-test or one-way ANOVA using the GraphPad Prism 6.01 software. The Chi-square test was also used wherever applicable. Data with *p* < 0.05 were considered significant.

## 3. Results

### 3.1. Cldn17^−/−^ Mice Exhibit Delayed Growth

**Figure 1:**
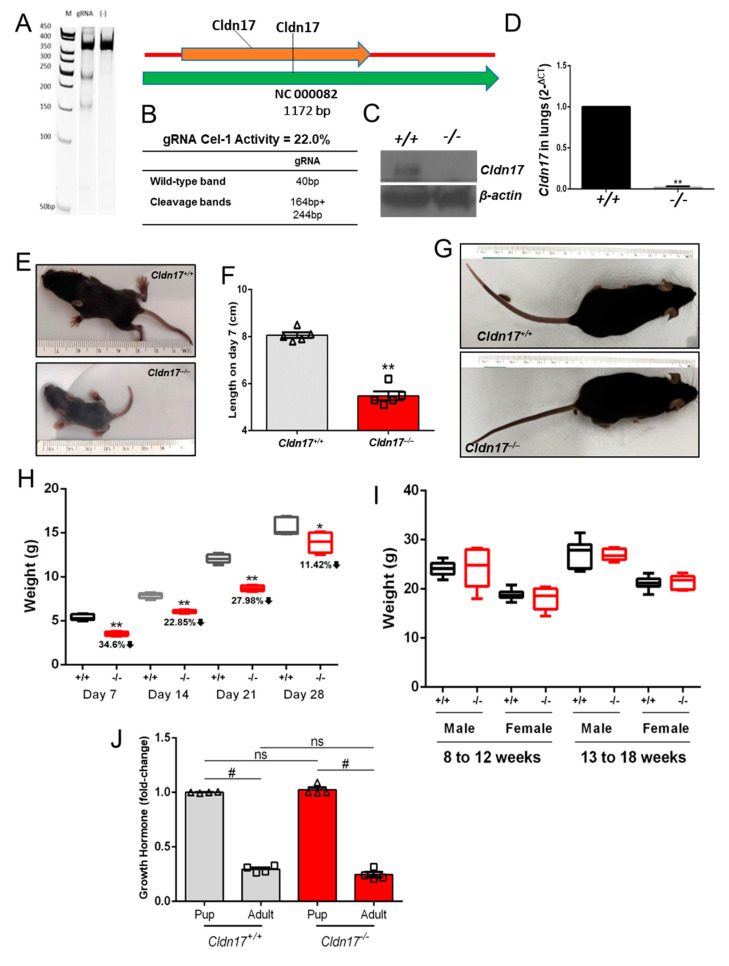
*Cldn17* deficiency delays growth in young mice. (**A**,**B**) Cel-1 assay validation gel representing *Cldn17* gene knockout and gRNA base-pair details for WT and cleavage bands. (**C**) Western blot images validating gene loss at the protein level in kidneys. (**D**) Bar graph showing mRNA level in lungs (*n* = 5). (**E**) Images (dorsal view) of WT and *Cldn17^−/−^* mice on day 7. (**F**) Lengths of WT and *Cldn17^−/−^* mice on day 7 (*n* =5). (**G**) Images (dorsal view) of WT and *Cldn17^−/−^* mice at week 14. (**H**) Growth hormone levels in pups and adult mice from WT and *Cldn17^−/−^* liver samples. (**I**) Weights of mice that were recorded at weeks 1 to 4 (*n* =5) and (**J**) weeks 8 to 18 (*n* =5–12). Data presented as mean ± s.e.m.; +/+, WT; –/–, *Cldn17* knockout; gRNA, guide RNA; bp, base-pair; g, gram; cm, centimeter; ns, not significant; * *p* < 0.05; ** *p* < 0.0001; # *p* < 0.01.

*Cldn17^−/−^* mice generated using the CRISPR/Cas9 technology were genotyped and further confirmed for gene loss by cell validation assay (Figure 1A,B), Western, and mRNA analysis in the kidney and the lung (Figure 1C,D). No breeding abnormalities were detected in the newly generated mice. To ascertain if *Cldn17* loss influences the growth of these mice, we recorded lengths and weights of the *Cldn17^−/−^* mice along with age- and gender-matched wild-type (WT) controls at various stages. Remarkably, *Cldn17^−/−^* mice appeared smaller than the WT controls on day 7 (Figure 1E), and the length measurements revealed a significant size reduction in mice lacking *Cldn17* (Figure 1F). Although the *Cldn17^−/−^* mice demonstrated a significant reduction in size also when measured at weeks 2 to 4, there was a gradual decline in the difference, and the *Cldn17^−/−^* mice reached parity with the WT at a later stage (Figure 1G), which was quantified by weights in the age range of 8 to 18 weeks involving both genders (Figure 1H,I). We also assessed the levels of growth hormones in the WT and the *Cldn17^−/−^* mice to determine if *Cldn17* has any control over this hormone. Liver lysates from both groups at early and late stages were subjected to ELISA to determine the levels of growth hormones. Although there was an expected reduction in the growth hormone level in the adult stage mice compared to the early-stage pups, no significant difference was observed between the WT and the *Cldn17^−/−^* mice (Figure 1J). Collectively, these findings signify that *Cldn17* plays a role in the overall growth of mice, at least in early life, however with no significant effect on growth hormone production.

### 3.2. Impact of Cldn17 Loss on Blood and Urine Parameters

**Figure 2:**
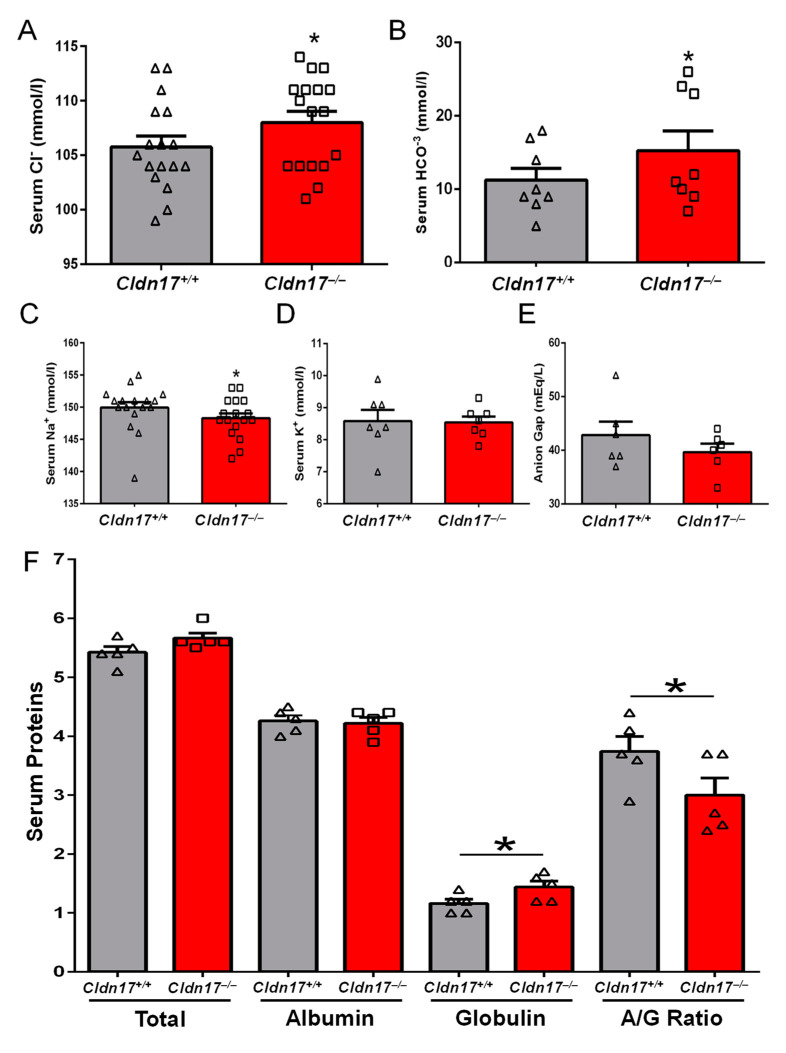
Loss of *Cldn17* affects blood electrolytes and proteins. Bar graphs showing variations in serum levels for (**A**) chloride (*n* = 17), (**B**) bicarbonates (*n* = 8), (**C**) sodium (*n* = 17), (**D**) potassium (*n* = 7), (**E**) anion gap (*n* = 6), and (**F**) proteins (total, albumin, globulin, and albumin/globulin ratio) in WT and *Cldn17^−/−^* mice. Data presented as mean ± s.e.m.; * *p* < 0.05.

Since *Cldn17* is a putative anionic pore-forming *CLDN* with a plausible role in ion homeostasis, we decided to analyze the levels of various electrolytes in serum samples obtained from WT and *Cldn17^−/−^* mice. Blood was collected through retro-orbital puncture before it was processed into serum and analyzed at the University of Georgia veterinary diagnostic laboratory. As anticipated, serum electrolytes were found to be altered in the *Cldn17^−/−^* mice with significantly elevated negatively charged chloride and bicarbonates (Figure 2A,B) and reduced levels of positively charged ions in the *Cldn17^−/−^* mice compared to WT (Figure 2C,D). A reduction in the anion gap was also observed in *Cldn17^−/−^* mice though the difference was not statistically significant (Figure 2E). In addition to the electrolytes, modulation in serum proteins was assessed in the absence of *Cldn17*. While total protein and albumin levels were unaltered in *Cldn17^−/−^* mice, total globulin and the albumin to globulin ratio were significantly reduced (Figure 2F). These results signify the vital role of *Cldn17* in ion and protein homeostasis in the blood.

Anticipating similar alterations in the urine, we collected urine from WT and *Cldn17^−/−^* mice using metabolic chambers for analysis. Interestingly, rare WBCs (white blood cells) presence in WT mice, considered normal, were significantly reduced in the *Cldn17^−/−^* mouse urine. Surprisingly, all other urine parameters such as the pH, specific gravity, color, bilirubin, bacteria, turbidity, and crystals showed no significant difference between WT and *Cldn17^−/−^* mice (Supplementary Figure S1).

### 3.3. Cldn17^−/−^ Mice Exhibit Hypophagia and Polydipsia

Given the retarded early growth in the absence of the *Cldn17* gene, we anticipated alterations in the food consumption in the genetically modified mice. We observed a significant reduction in the food consumption in *Cldn17^−/−^* mice against the control (Supplementary Figure S2A). Since *Cldn17* is a TJ protein expressed in the proximal tubule responsible for water reabsorption in the kidneys, we also determined water intake, which revealed significantly increased water consumption by *Cldn17^−/−^* mice compared to the WT (Supplementary Figure S2B). Analysis of fecal pellets revealed a significant reduction in the number of pellets excreted over 24 h by *Cldn17^−/−^* mice compared to the WT group (Supplementary Figure S2C). Wet/dry ratio analysis of these fecal pellets was also performed, which revealed a significantly lower moisture content in the fecal pellets collected from mice lacking *Cldn17* (Supplementary Figure S2D,E), signifying constipation-like effects in *Cldn17^−/−^* mice.

### 3.4. Cldn17^−/−^ Mice Display Kidney Injury

**Figure 3:**
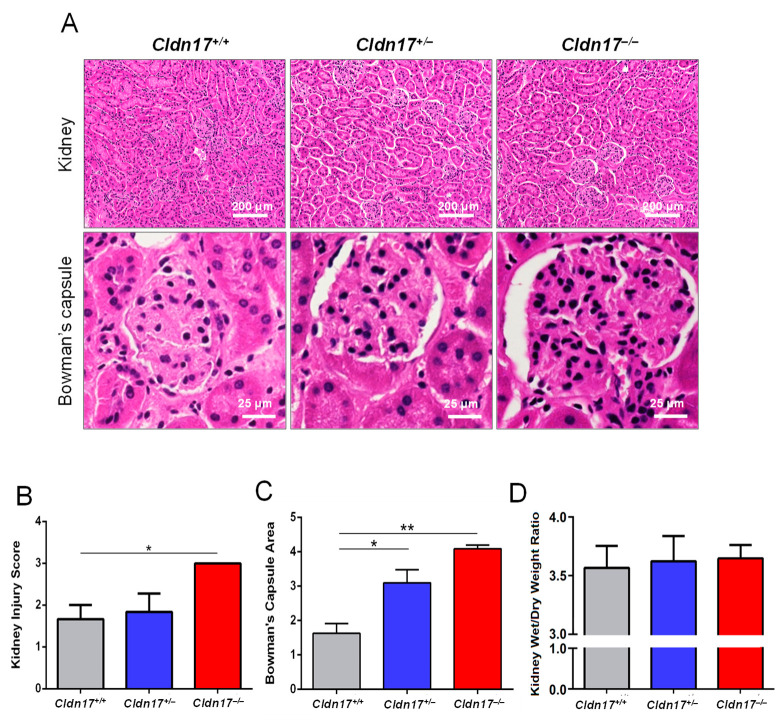
*Cldn17^−/−^* mice display kidney injury. (**A**) Hematoxylin and eosin-stained images of kidneys with magnified Bowman’s capsule area from WT, *Cldn17^+/−^*, and *Cldn17^−/−^* mice. (**B**) Bar graph showing kidney injury score determined by blinded reviewers and quantification of renal edema by wet/dry ratio. (**C**) Bar graph showing quantification of Bowman’s capsule area by Image J software. (**D**) Bar graph showing quantification of renal edema by wet/dry ratio. *n* = 3–5; * *p* < 0.05; ** *p* < 0.01.

Age, gender, and weight-matched WT, *Cldn17^+/−^*, and *Cldn17^−/−^* mice were euthanized, and their kidneys were collected, sectioned, and subjected to H&E staining. Since *Cldn17* is expressed in the kidneys, a potential role of *Cldn17* in kidney function is expected. As anticipated, there was increased injury in the *Cldn17^+/−^* and *Cldn17^−/−^* mouse kidneys compared to WT, characterized by vacuolar degeneration, mineralization, and increased Bowman’s capsule spacing (Figure 3A). Kidney injury scoring was performed by blinded reviewers on a scale of 1–5 revealed a higher injury in the *Cldn17^−/−^* mice compared to WT.

(Figure 3B and Supplementary Figure S3A,B). Further, ImageJ software quantification of the area of Bowman’s capsule revealed significantly enlarged capsule size in *Cldn17^−/−^* mice compared to WT (Figure 3C). Wet/dry ratio analysis assessed renal edema which showed no significant difference between the two groups (Figure 3D and Supplementary Figure S3A–C). Although *Cldn17* is highly expressed in the keratinocytes [33] and hepatocytes [26], we did not observe any abnormalities in the histological examinations of skin and liver sections from *Cldn17^+/+^* and *Cldn17^−/−^* mice (Supplementary Figure S4).

### 3.5. Cldn17 Loss Affects the Expression of other Cldns in Mouse Kidneys

**Figure 4:**
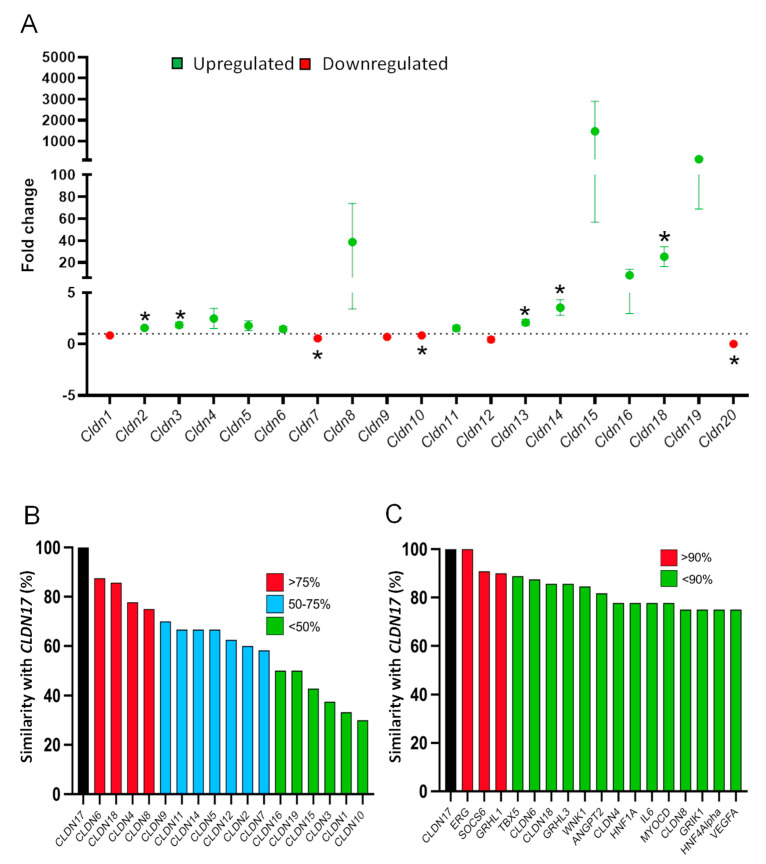
Loss of *Cldn17* affects the expression of a few other *Cldn* isoforms. (**A**) Bar graph showing changes in the expression of other *Cldns* in *Cldn17^-/-^* mouse kidneys compared to WT as analyzed by quantitative RT-PCR analysis. *n* = 3; * *p* < 0.05. (**B**) Bar graph showing GEO database analysis of other *CLDN* genes that are coregulated in various physiological and pathological events presented in percent coregulation. (**C**) Bar graph showing GEO database analysis of all other genes that are co-regulated in various physiological and pathological events presented in percent coregulation.

Quantitative RT-PCR analysis of mouse kidneys revealed a significant upregulation of *Cldn-2*, *-3*, *-13*, *-14*, and *-18* (Figure 4A). Interestingly, a downregulation in the expression of *Cldn-7*, *-10*, and -*20* was also observed (Figure 4A). Although not significant, the highest increase in expression (likely due to high variability between the samples) was observed in *Cldn-4-15,* and *-19*, all of which are cation pore-forming *Cldns*.

### 3.6. CLDN17 Co-Expression with Other Genes in Physiology and Pathology

Among all *CLDNs*, expression of *CLDN-6, -18, -4,* and *-8* demonstrated a higher (<75%) resemblance to *CLDN17* trends upon intervention (Figure 4B). Apart from the CLDN family, a few genes revealed higher similarity with *CLDN17* including the ETS-related gene (ERG) with 100% resemblance. Interestingly, the suppressor of cytokine signaling (SOCS6) and grainy head-like transcription factor-1 (GRHL1) demonstrated *CLDN17*-like in 90% of interventions (Figure 4C).

### 3.7. RNA-Seq Analysis Revealed Alterations in Genes Involved in Inflammation, Hypoxia, and ROS Generation

**Figure 5:**
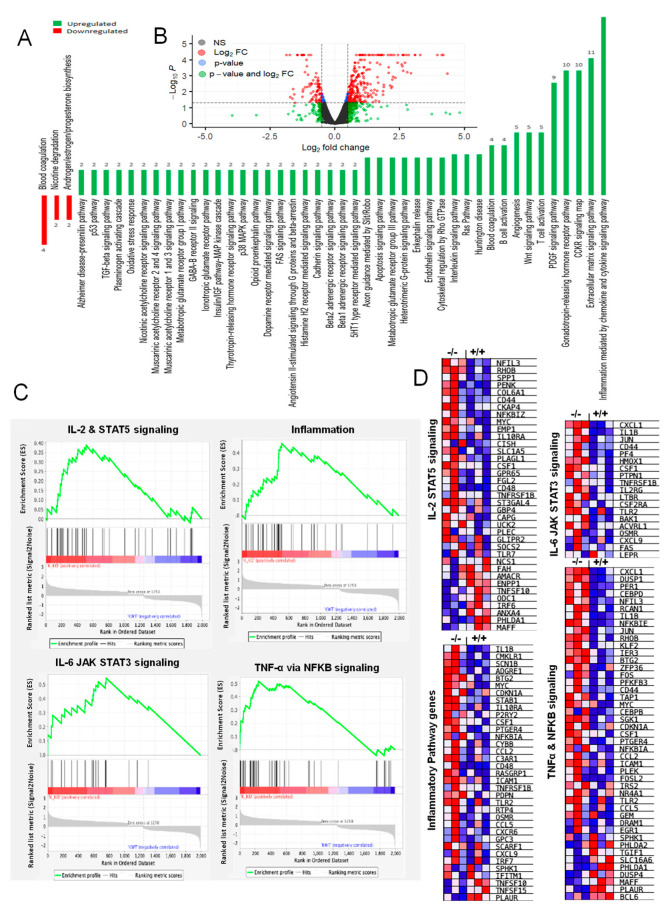
RNA-seq analysis reveals altered gene expression in *Cldn17^−/−^* mouse kidneys. (**A**) Alteration in signaling pathways in the *Cldn17^−/−^* mice against the WT group based on the Panther database. (**B**) Distribution of genes between WT and *Cldn17^−/−^* mice on a volcano plot. (**C**) Gene enrichment plots (GO) and (**D**) heat maps (GSEA) for altered genes involved in the regulation of inflammation. Data represented for genes with fold-change >2; *n* = 3; GO, gene ontology; GSEA, gene set enrichment analysis; NS, not significant; FC, fold-change; IL, interleukin; TNF, tumor necrosis factor; +/+, WT; −/−, *Cldn17* knockout; *p* < 0.05.

A series of intriguing observations from the *Cldn17^−/−^* mice prompted us to perform RNA-seq analysis of their kidneys. Analysis of 12,031 genes in WT and *Cldn17^−/−^* mouse kidneys revealed significant changes in 424 genes, with 295 upregulations and 119 downregulations in gene expression in the *Cldn17^−/−^* group compared to WT (Figure 5, Supplementary Figure S5). Interestingly, *Cldn17^−/−^* kidneys had *de novo* expression of ten genes that are not expressed in the WT kidneys.

**Figure 6:**
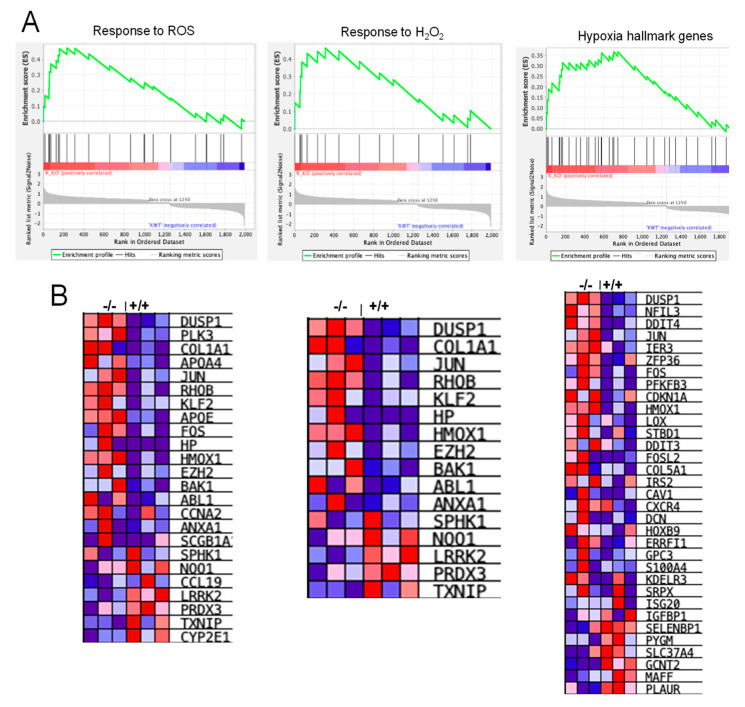
*Cldn17* loss modulates the expression of genes regulating oxidative stress. (**A**) Gene enrichment plots (GO) and (**B**) heat maps (GSEA) depicting modulation of genes related to ROS, H_2_O_2_, and hypoxia in kidney samples from *Cldn17^−/−^* mice against the WT group on RNA sequencing. Data represented for genes with fold-change >2; *n* =3.

Next, we identified the most deregulated signaling pathways using the Panther database (Figure 5A,B). The top three downregulated pathways were associated with coagulation, nicotine degradation, and androgen/estrogen/progesterone biosynthesis-related signaling. In contrast, upregulated genes were linked to inflammation, integrins (cell adhesion and migration), angiogenesis, lipid metabolism, and gonadotropin hormone receptor signaling. In the next step, RNA-seq data were subjected to gene ontology (GO) and gene set enrichment analysis (GSEA) analyses based on genes modulated more than two-fold. From the highly influenced signaling pathways, we identified some pathways of interest based on our study objectives. Interestingly, inflammatory pathways, including TNFα and IL-related signaling, were enhanced in the *Cldn17^−/−^* mice compared to WT (Figure 5C). Furthermore, we observed modulations in the expression of genes regulating the response to reactive oxygen species, H_2_O_2_, and hypoxia (Figure 6A,B).

### 3.8. Cldn17 Loss Did Not Affect the Expression of Vasopressin and Angiotensin Pathway Genes in Mice

Next, we determined if changes in the water content in feces are associated with any changes in the expression of genes regulating the vasopressin and angiotensin pathway. Analysis of kidney RNA-seq data revealed no significant changes in any genes involved in this pathway between Cldn17^+/+^ and Cldn17^−/−^ mice (Supplementary Tables S1 and S2).

### 3.9. Cldn17 Deficiency Promotes ROS Generation in the Kidneys

**Figure 7:**
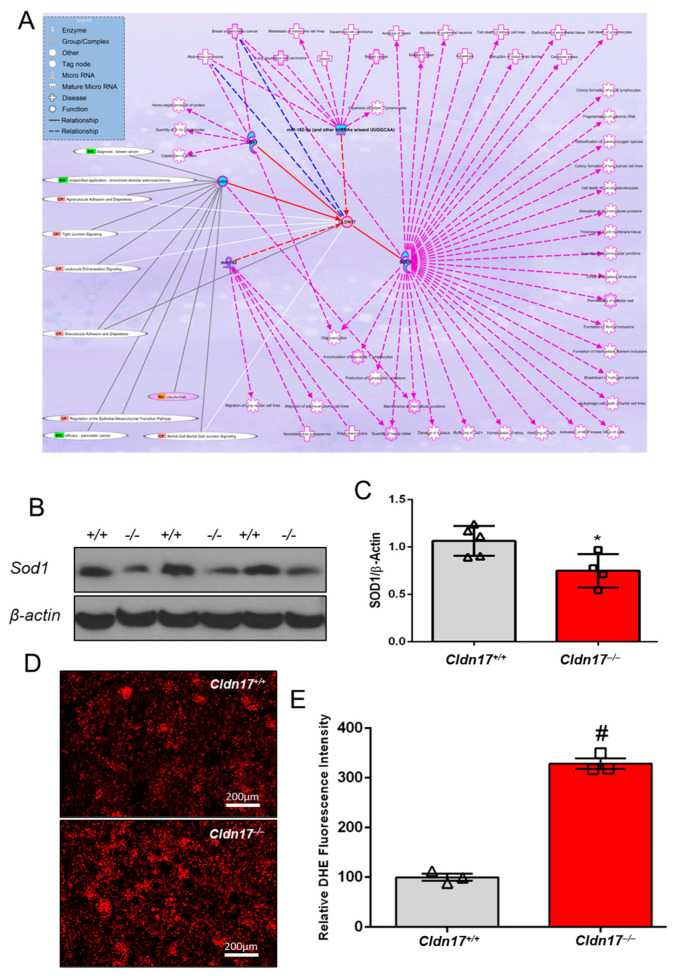
*Cldn17* loss modulates SOD1 expression resulting in increased reactive oxygen species levels in the kidneys. (**A**) Ingenuity pathway analysis predicting a potential link between *CLDN17* and SOD1. (**B**) Representative images of immunoblots and (**C**) densitometry analysis of SOD1 expression in kidney lysates obtained from WT and *Cldn17^−/−^* mice. (**D**) DHE-stained images and (**E**) fluorescence quantification of ROS on frozen kidney sections from both groups of mice. Data presented as mean ± s.e.m.; GO, gene ontology; GSEA, gene set enrichment analysis; ROS, reactive oxygen species; H_2_O_2_, hydrogen peroxide; SOD1, superoxide dismutase1; DHE, dihydroethidium; *p < 0.05; # *p* < 0.01.

**Figure 8:**
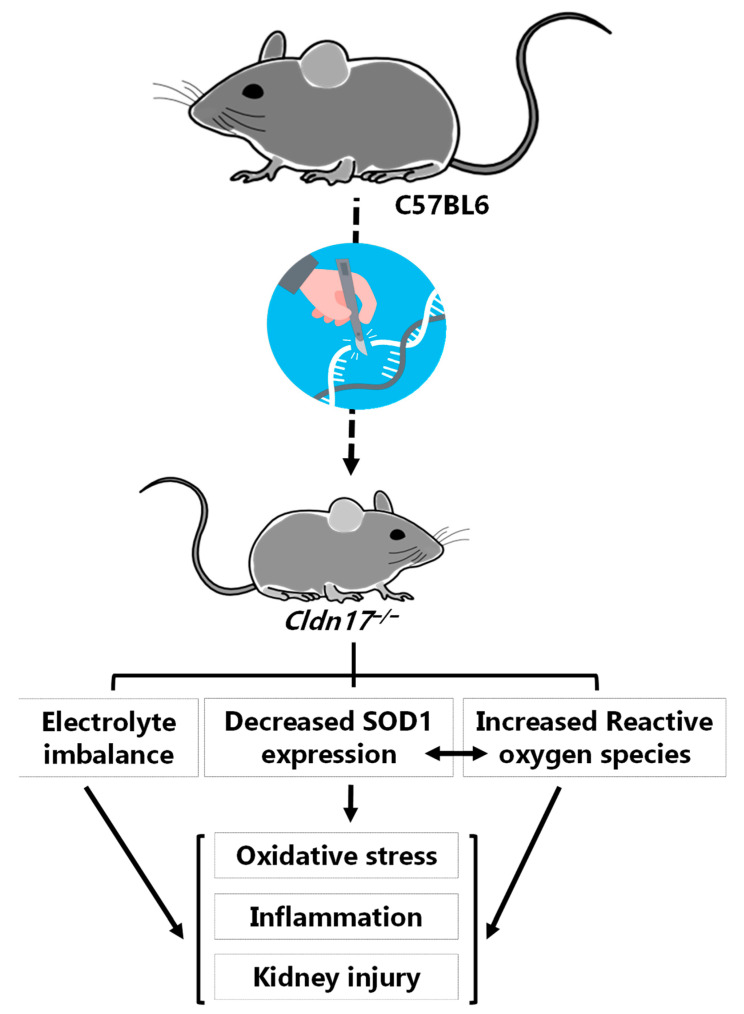
*Cldn17* deficiency results in kidney injury as a result of an imbalance in electrolytes and increased ROS levels associated with reduced expression of SOD1 and modulation of a few other genes that are involved in ROS generation, leading to oxidative stress, inflammation, and kidney injury in mice. Pharmacological interventions to prevent the loss of *Cldn17* or increase its expression may have therapeutic benefits for kidney and other organ injuries.

Since ROS are charged molecules, *Cldn17* may be potentially involved in their clearance from tissues. Ingenuity pathway analysis (IPA) suggested a potential link between *CLDN17* and *SOD1* (Figure 7A and Supplementary Figure S6), which was confirmed by Western blot analysis of kidney lysates, revealing significantly lower *Sod1* expression in *Cldn17^−/−^* kidneys (Figure 7B,C). In addition, dihydroethidium (DHE) staining of frozen kidney sections indicated increased ROS levels in *Cldn17^−/−^* kidneys compared to WT (Figure 7D,E and Figure 8).

## 4. Discussion

Defects in endothelial and epithelial cell-barrier TJs cause perturbations in vascular homeostasis leading to blood–tissue-barrier damage, inflammation, and injury to organs [5,34]. Among the various TJ proteins, the 27-gene member family of *CLDNs* plays the most significant role in blood-tissue barrier regulation and tissue integrity [17,35]. Whereas several *CLDNs* have been studied in epithelial cells (EpC) [1,17], *CLDN5* is the only well-characterized member in ECs [13,14]. Intriguingly, the blood-tissue barrier is intact in *Cldn5^−/−^* mice except for a modest and selective leakage of very low molecular weight proteins in the brain [36]. There is a significant gap in our knowledge of the expression, mechanisms, and role of various *CLDNs* in ECs and blood-tissue barriers in various organs. The Akt1-FoxO-βCatenin pathway has been demonstrated to regulate vascular permeability by modulating the expression of TJ *CLDNs* [14,15,37,38]. Although its significance is not clear, we observed a correlation between loss of *CLDN17*, a putative anion pore-forming *CLDN* [19,39], with aberrant EC-barrier disruption [14]. Until today, the role of *Cldn17* in physiology and pathology in vivo has not been investigated, except for a few conflicting reports on its deficiency linking to several cancers. In the current study, our results demonstrate a vital role for *Cldn17* in early development, regulation of electrolyte homeostasis, ROS generation, and inflammation in kidney injury. Despite the early delay in their growth, *Cldn17^−/−^* mice reached parity with WT mice in their weight and length and exhibited no changes in growth hormone levels, breeding abnormalities, or mortality until 18 months of observation. While the exact reasons for this growth lag are not clear, the literature indicates a similar phenotype in mice lacking the *Ocln* gene, another TJ protein, where a size reduction was observed that subsided by 8 weeks [40].

*Cldn17* is an intron-less gene reported to be primarily expressed in the kidneys and brain [17,22,39]. *Cldn17* is a paracellular anion channel that is permeable for halides, HCO_3_–, NO_3_–, and organic anions [19,39] suggesting a potential role in buffering, electrolyte, and fluid balance across the cell barrier. An increase in serum chloride and bicarbonate levels accompanied by a decrease in sodium and potassium levels and a reduced anion gap in *Cldn17^−/−^* mice compared to WT is a first-time report signifying the importance of *Cldn17^−/−^* in ion, electrolyte, and pH homeostasis. It is confirmation of its anticipated role based on the structural evidence. An increase in serum globulin levels and a decreased albumin to globulin ratio in *Cldn17^−/−^* mice vs. WT further confirms this conclusion. Furthermore, our observations on the anion-channel property of *Cldn17* were corroborated with reduced symporter activity in an RNA-seq analysis in *Cldn17^−/−^* mice. Surprisingly, no significant proteinuria was observed in *Cldn17^−/−^* mice. Since chloride channel activation regulates fluid balance in the intestines helping with the smooth passage of stools [41], reduced number of fecal pellets in the excreta and reduced fecal water content in *Cldn17^−/−^* mice compared to WT controls could also be a result of chloride imbalance in *Cldn17^−/−^* mice.

The findings from RNA-seq analysis further reinforced our notion that systemic inflammation accompanies *Cldn17* deficiency. This coincides with a clinical study linking altered *CLDN17* expression to adhesion and diapedesis of agranulocytes and granulocytes, leading to bronchopulmonary dysplasia, a chronic lung disease [42]. Another clinical study reported a significant upregulation of *CLDN17* in the biopsy samples from oral lichen planus, a chronic, T cell-mediated inflammatory, autoimmune mucosal disease [43], thus suggesting a potential role for *CLDN17* in inflammation. Furthermore, IPA analysis and the RNA-seq data revealed the potential involvement of ROS in inflammation and organ injuries observed in *Cldn17^−/−^* mice. These analyses established a robust relationship between *CLDN17* and pathways linked to oxygen homeostasis. IPA data predicated a potential link between *CLDN17* and *SOD1*, a potent regulator of ROS levels in tissues [44]. As predicted, *Sod1* levels were significantly reduced in *Cldn17^−/−^* kidneys accompanied by increased ROS levels in *Cldn17^–/−^* kidneys, evidenced by DHE staining. Moreover, RNA-seq revealed increased expression of genes regulating oxidative stress and cytochrome P450 enzymes, which have been indicated in ROS generation [45].

Although the current study identifies an important role for *Cldn17* in ion and electrolyte homeostasis, and the regulation of ROS generation resulting in mild damage to the kidneys, the study has some limitations. The kidney injury observed in *Cldn17^−/−^* mice is mild, which could be because no external insult were used, and the comparisons are made between the un-injured WT and *Cldn17^−/−^* mouse kidneys. Kidney injury has only been demonstrated by the injury score, Bowman’s capsule area analysis, and ROS levels in the kidney sections and not kidney function tests. Our study also did not reveal any significant changes in the urine parameters such as the specific gravity, color, cloudiness, bacterial content, number of blood cells, protein, bilirubin, or pH between the WT and *Cldn17^−/−^* mice. The observed changes in ions and proteins were limited to the plasma. Future studies focused on experimental models of induced-kidney injury and kidney function tests will be needed for deeper characterization of the role of *Cldn17* in kidneys and other organs.

## 5. Conclusions

Altogether, the observations from the current study, including the data from gene expression profiling, collectively suggest a regulatory role of *CLDN17* in many physiological mechanisms that include maintenance of blood-tissue barrier integrity and ion, electrolyte, and ROS homeostasis (Figure 8). Further, deficiency of *Cldn17* in mice leads to negative effects, including but not limited to inflammation and kidney damage.

## Data Availability

The datasets generated and analyzed during the current study are available from the corresponding author upon reasonable request. Source data are provided in this paper.

## Supplementary Materials

The following supporting information can be downloaded at: https://www.mdpi.com/article/10.3390/cells11111782/s1, Figure S1: Urinalysis of samples from WT and Cldn17 null animals; Figure S2: Effect of Cldn17 loss in mice on water balance; Figure S3: Cldn17^−/−^ mice display kidney injury; Figure S4: Effect of Cldn17 loss on skin and liver; Figure S5: The butterfly plot for modulated genes in WTs and Cldn17 knockout groups; Figure S6: Ingenuity pathway analysis predicting a potential link between CLDN17 and SOD1; Figure S7: Identification of wild-type, heterozygote, and homozygote animals with small deletions (1–200 bp); Table S1: No changes in the expression of vasopressin genes in the kidneys of Cldn17^−/−^ mice (*n* = 3); Table S2: No changes in the expression of Angiotensin genes in the kidneys of Cldn17^−/−^ mice (*n* = 3); Table S3: RT-PCR Primers for mouse Claudins.

## References

[1] Günzel D. (2017). Claudins: Vital partners in transcellular and paracellular transport coupling. Pflug. Arch..

[2] Elkouby-Naor L., Ben-Yosef T. (2010). Functions of claudin tight junction proteins and their complex interactions in various physiological systems. Int. Rev. Cell Mol. Biol.

[3] Shekarabi M., Zhang J., Khanna A.R., Ellison D.H., Delpire E., Kahle K.T. (2017). WNK Kinase Signaling in Ion Homeostasis and Human Disease. Cell Metab.

[4] Kawedia J.D., Nieman M.L., Boivin G.P., Melvin J.E., Kikuchi K., Hand A.R., Lorenz J.N., Menon A.G. (2007). Interaction between transcellular and paracellular water transport pathways through Aquaporin 5 and the tight junction complex. Proc. Natl. Acad. Sci. USA.

[5] Park-Windhol C., D’Amore P.A. (2016). Disorders of Vascular Permeability. Annu. Rev. Pathol..

[6] Wen Q., Tang E.I., Gao Y., Jesus T.T., Chu D.S., Lee W.M., Wong C.K.C., Liu Y.X., Xiao X., Silvestrini B. (2018). Signaling pathways regulating blood-tissue barriers-Lesson from the testis. Biochim. Biophys Acta Biomembr..

[7] Frank J.A. (2012). Claudins and alveolar epithelial barrier function in the lung. Ann. N. Y. Acad. Sci..

[8] Bhattad P.B., Jain V. (2020). Renal Artery Stenosis As Etiology of Recurrent Flash Pulmonary Edema and Role of Imaging in Timely Diagnosis and Management. Cureus.

[9] Owen-Woods C., Joulia R., Barkaway A., Rolas L., Ma B., Nottebaum A.F., Arkill K.P., Stein M., Girbl T., Golding M. (2020). Local microvascular leakage promotes trafficking of activated neutrophils to remote organs. J. Clin. Investig..

[10] Lee K.S., Kim S.R., Park S.J., Park H.S., Min K.H., Lee M.H., Jin S.M., Jin G.Y., Yoo W.H., Lee Y.C. (2006). Hydrogen peroxide induces vascular permeability via regulation of vascular endothelial growth factor. Am. J. Respir Cell Mol. Biol..

[11] Incalza M.A., D’Oria R., Natalicchio A., Perrini S., Laviola L., Giorgino F. (2018). Oxidative stress and reactive oxygen species in endothelial dysfunction associated with cardiovascular and metabolic diseases. Vasc. Pharm..

[12] Ikari A., Atomi K., Yamazaki Y., Sakai H., Hayashi H., Yamaguchi M., Sugatani J. (2013). Hyperosmolarity-induced up-regulation of claudin-4 mediated by NADPH oxidase-dependent H2O2 production and Sp1/c-Jun cooperation. Biochim. Biophys. Acta (BBA)-Mol. Cell Res..

[13] Taddei A., Giampietro C., Conti A., Orsenigo F., Breviario F., Pirazzoli V., Potente M., Daly C., Dimmeler S., Dejana E. (2008). Endothelial adherens junctions control tight junctions by VE-cadherin-mediated upregulation of claudin-5. Nat. Cell Biol..

[14] Gao F., Artham S., Sabbineni H., Al-Azayzih A., Peng X.D., Hay N., Adams R.H., Byzova T.V., Somanath P.R. (2016). Akt1 promotes stimuli-induced endothelial-barrier protection through FoxO-mediated tight-junction protein turnover. Cell Mol. Life Sci..

[15] Gao F., Sabbineni H., Artham S., Somanath P.R. (2017). Modulation of long-term endothelial-barrier integrity is conditional to the cross-talk between Akt and Src signaling. J. Cell Physiol..

[16] Alwhaibi A., Verma A., Adil M.S., Somanath P.R. (2019). The unconventional role of Akt1 in the advanced cancers and in diabetes-promoted carcinogenesis. Pharm. Res..

[17] Adil M.S., Narayanan S.P., Somanath P.R. (2020). Cell-cell junctions: Structure and regulation in physiology and pathology. Tissue Barriers.

[18] Rosenthal R., Günzel D., Theune D., Czichos C., Schulzke J.D., Fromm M. (2017). Water channels and barriers formed by claudins. Ann. N. Y. Acad. Sci..

[19] Conrad M.P., Piontek J., Günzel D., Fromm M., Krug S.M. (2016). Molecular basis of claudin-17 anion selectivity. Cell Mol. Life Sci..

[20] Marcelino Cereijido J.M.A. (2001). Tight Junctions.

[21] Shabalina S.A., Ogurtsov A.Y., Spiridonov A.N., Novichkov P.S., Spiridonov N.A., Koonin E.V. (2010). Distinct patterns of expression and evolution of intronless and intron-containing mammalian genes. Mol. Biol. Evol..

[22] Günzel D., Yu A.S. (2013). Claudins and the modulation of tight junction permeability. Physiol Rev..

[23] Kielgast F., Schmidt H., Braubach P., Winkelmann V.E., Thompson K.E., Frick M., Dietl P., Wittekindt O.H. (2016). Glucocorticoids Regulate Tight Junction Permeability of Lung Epithelia by Modulating Claudin 8. Am. J. Respir Cell Mol. Biol..

[24] Li Z.H., Xia T.H., Kang Z.J., Deng X., Wang Y. (2018). Expression and significance of tight junction proteins in the kidney in a mouse model of renal ischemia-reperfusion injury. Zhongguo Dang Dai Er Ke Za Zhi.

[25] Xu Y.N., Deng M.S., Liu Y.F., Yao J., Xiao Z.Y. (2022). Tight junction protein CLDN17 serves as a tumor suppressor to reduce the invasion and migration of oral cancer cells by inhibiting epithelial-mesenchymal transition. Arch Oral Biol..

[26] Sun L., Feng L., Cui J. (2018). Increased expression of claudin-17 promotes a malignant phenotype in hepatocyte via Tyk2/Stat3 signaling and is associated with poor prognosis in patients with hepatocellular carcinoma. Diagn Pathol..

[27] Gao M., Li W., Wang H., Wang G. (2013). The distinct expression patterns of claudin-10, -14, -17 and E-cadherin between adjacent non-neoplastic tissues and gastric cancer tissues. Diagn Pathol..

[28] Berndt P., Winkler L., Cording J., Breitkreuz-Korff O., Rex A., Dithmer S., Rausch V., Blasig R., Richter M., Sporbert A. (2019). Tight junction proteins at the blood-brain barrier: Far more than claudin-5. Cell Mol. Life Sci..

[29] Alharthi A., Verma A., Sabbineni H., Adil M.S., Somanath P.R. (2021). Distinct effects of pharmacological inhibition of stromelysin1 on endothelial-to-mesenchymal transition and myofibroblast differentiation. J. Cell Physiol..

[30] Gah A., Adil M.S., Sabbineni H., Verma A., Somanath P.R. (2020). Differential regulation of TGFβ type-I receptor expressions in TGFβ1-induced myofibroblast differentiation. Can J. Physiol. Pharm..

[31] Krämer A., Green J., Pollard J., Tugendreich S. (2014). Causal analysis approaches in Ingenuity Pathway Analysis. Bioinformatics.

[32] Wang Q., Zou M.H. (2018). Measurement of Reactive Oxygen Species (ROS) and Mitochondrial ROS in AMPK Knockout Mice Blood Vessels. Methods Mol. Biol..

[33] Brandner J.M., Kief S., Grund C., Rendl M., Houdek P., Kuhn C., Tschachler E., Franke W.W., Moll I. (2002). Organization and formation of the tight junction system in human epidermis and cultured keratinocytes. Eur. J. Cell Biol..

[34] Cong X., Kong W. (2020). Endothelial tight junctions and their regulatory signaling pathways in vascular homeostasis and disease. Cell. Signal..

[35] Rudraraju M., Narayanan S.P., Somanath P.R. (2020). Regulation of blood-retinal barrier cell-junctions in diabetic retinopathy. Pharm. Res..

[36] Nitta T., Hata M., Gotoh S., Seo Y., Sasaki H., Hashimoto N., Furuse M., Tsukita S. (2003). Size-selective loosening of the blood-brain barrier in claudin-5-deficient mice. J. Cell Biol..

[37] Chen J., Somanath P.R., Razorenova O., Chen W.S., Hay N., Bornstein P., Byzova T.V. (2005). Akt1 regulates pathological angiogenesis, vascular maturation and permeability in vivo. Nat. Med..

[38] Gao F., Alwhaibi A., Artham S., Verma A., Somanath P.R. (2018). Endothelial Akt1 loss promotes prostate cancer metastasis via beta-catenin-regulated tight-junction protein turnover. Br. J. Cancer.

[39] Krug S.M., Günzel D., Conrad M.P., Rosenthal R., Fromm A., Amasheh S., Schulzke J.D., Fromm M. (2012). Claudin-17 forms tight junction channels with distinct anion selectivity. Cell Mol. Life Sci..

[40] Runkle E.A., Mu D. (2013). Tight junction proteins: From barrier to tumorigenesis. Cancer Lett..

[41] Portalatin M., Winstead N. (2012). Medical management of constipation. Clin. Colon. Rectal Surg..

[42] Torgerson D.G., Ballard P.L., Keller R.L., Oh S.S., Huntsman S., Hu D., Eng C., Burchard E.G., Ballard R.A. (2018). Ancestry and genetic associations with bronchopulmonary dysplasia in preterm infants. Am. J. Physiol Lung Cell Mol. Physiol..

[43] Chen J., Du G., Wang Y., Shi L., Mi J., Tang G. (2017). Integrative analysis of mRNA and miRNA expression profiles in oral lichen planus: Preliminary results. Oral Surg. Oral Med. Oral Pathol. Oral Radiol..

[44] Bunton-Stasyshyn R.K., Saccon R.A., Fratta P., Fisher E.M. (2015). SOD1 Function and Its Implications for Amyotrophic Lateral Sclerosis Pathology: New and Renascent Themes. Neuroscientist.

[45] Veith A., Moorthy B. (2018). Role of cytochrome p450s in the generation and metabolism of reactive oxygen species. Curr. Opin. Toxicol..

