# Supplementary material for: Claudin-17 Deficiency in Mice Results in Kidney Injury Due to Electrolyte Imbalance and Oxidative Stress
Source: Cells. 2022 May 29;11(11):1782. doi: 10.3390/cells11111782 (PMC9180152; doi:10.3390/cells11111782)
Supplement: Supplementary file 1 [file cells-11-01782-s001.zip › cells-1675192-supplementary.pdf]

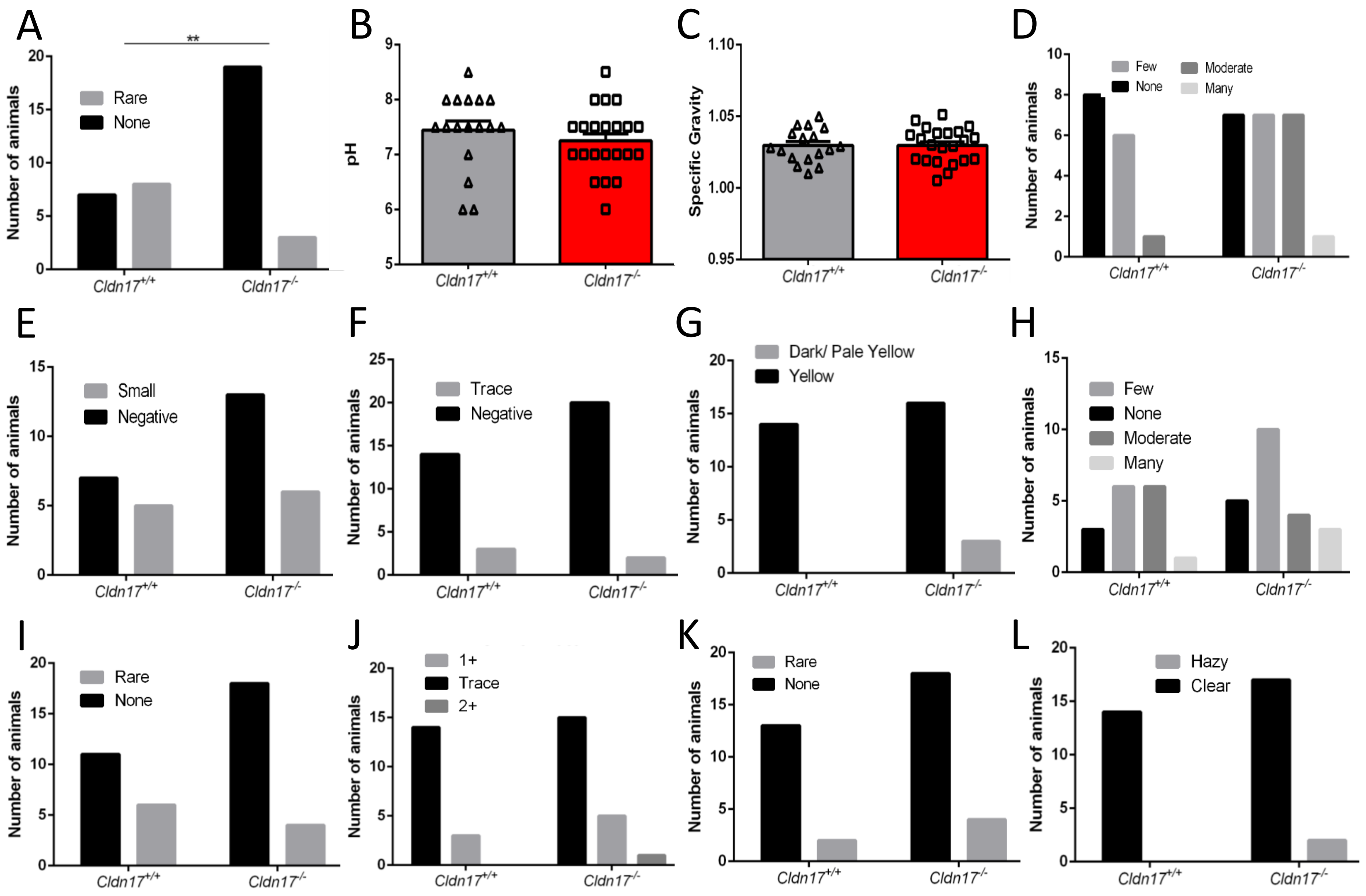

Supplementary Figure S1. Urinalysis of samples from WT and Cldn17 null animals. Bar graphs showing changes in **A.** WBC (n=15-22), **B.** pH (n=17-22), **C.** Specific gravity (n=17-22), **D.** Bacteria (n=15-22), **E.** Bilirubin (n=12-19), **F.** Blood (n=17-22), **G.** Color (n=14-19), **H.** Crystals (n=16-22), **I.** Epithelial cells (n=17-22), **J.** Protein (n=14-19), **K.** RBC (n=15-22), **L.** Turbidity (n=14-19) in mouse urine. **p<0.01.

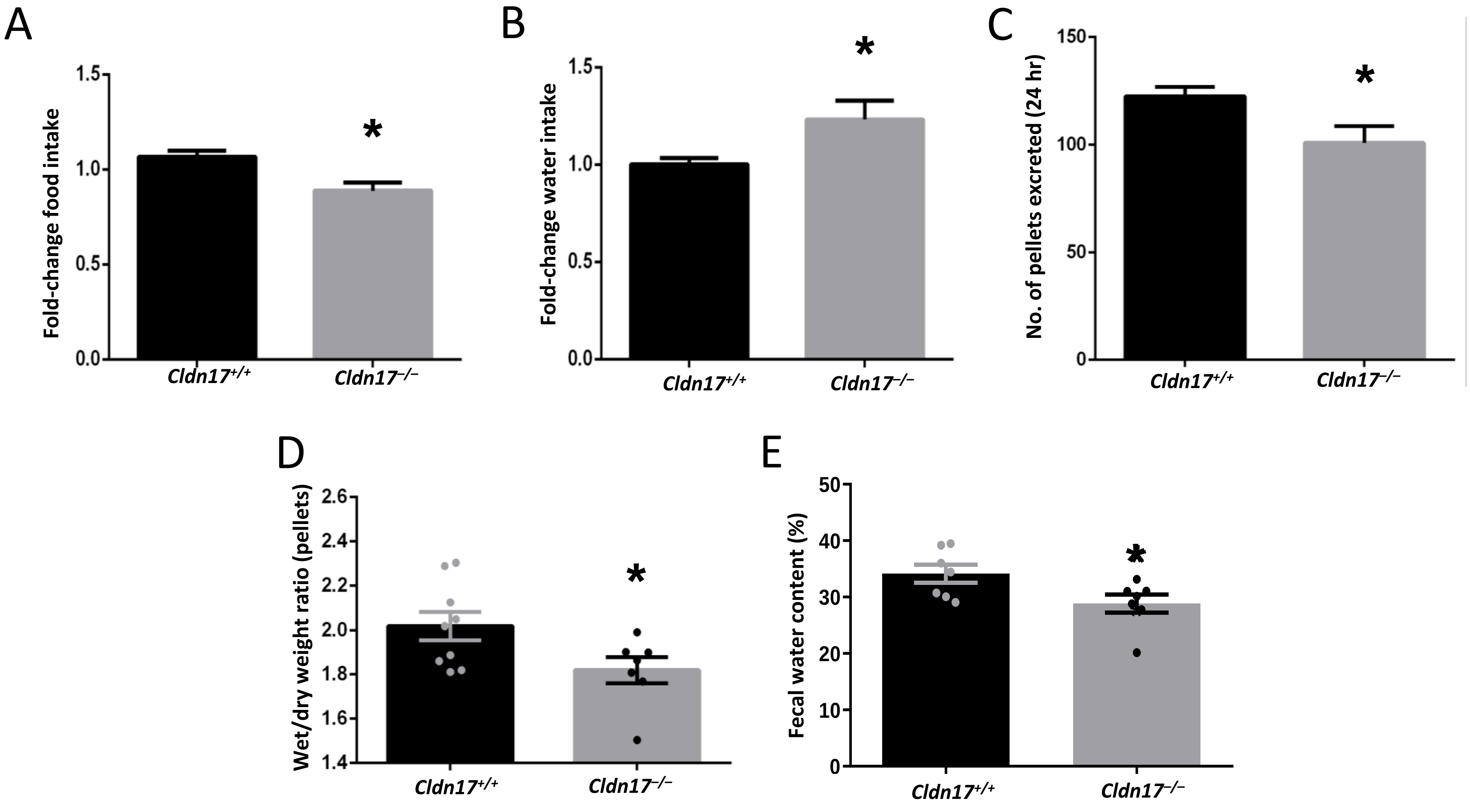

Supplementary FigureS2. Effect of *Cldn17* loss in mice on water balance. **A** Fold-change food (n=8) and **B** Fold-change water (n=9) consumption in WT and *Cldn17*$^{-/-}$ mice. **C** Fecal pellets excreted over 24 hours (n=4 cages, 2-4 mice per cage) from WT and *Cldn17*$^{-/-}$ mice. **D** Wet/dry ratio of fecal pellets demonstrating the moisture content (n=7-9). **E** Water content in the fecal pellets from WT and *Cldn17*$^{-/-}$ mice (n=7). Data presented as mean ± s.e.m.; *p<0.05.

A

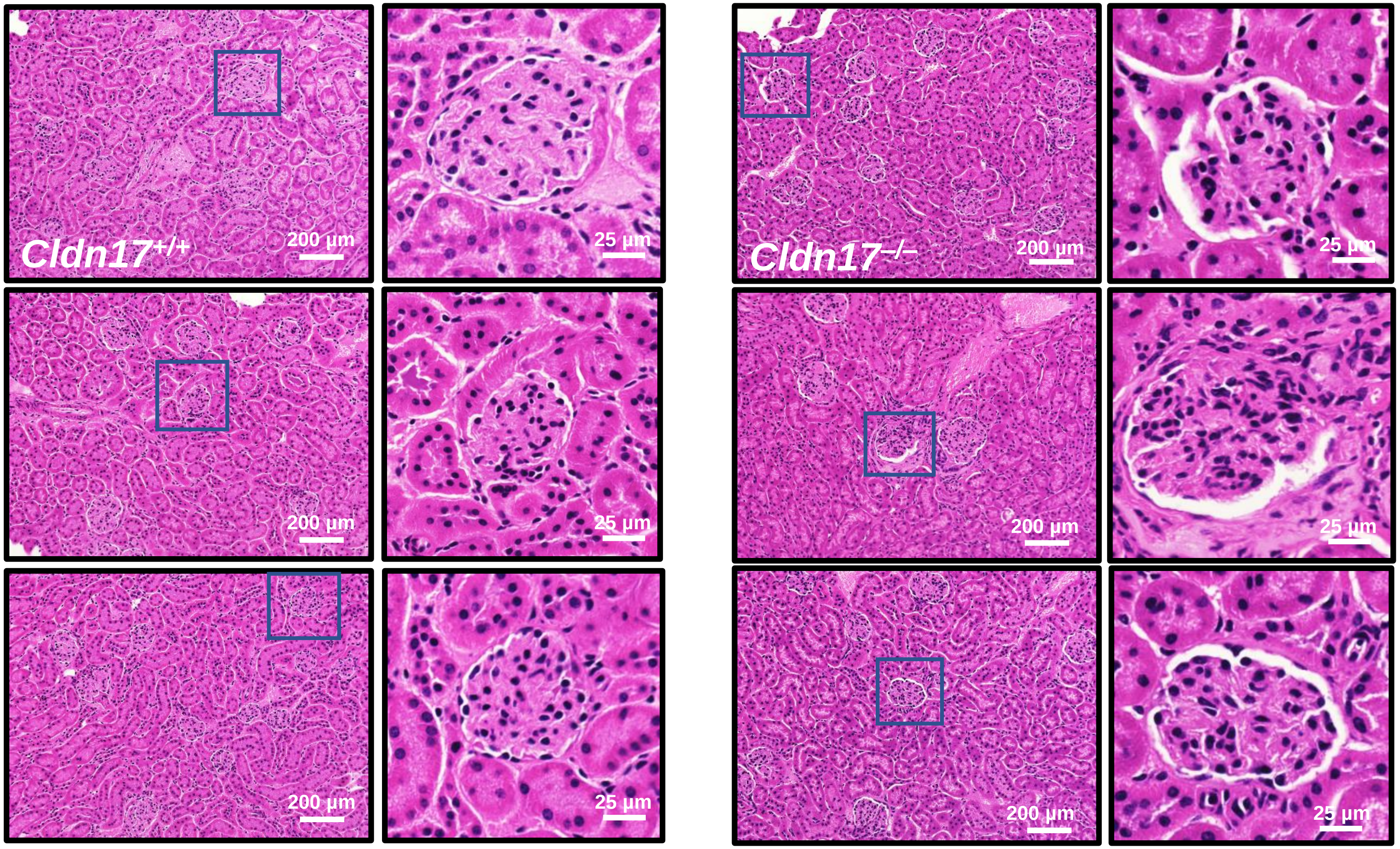

B

Injury scores (average values of 3 fields/mouse)			
	Cldn17$^{+/+}$	*Cldn17*$^{+/-}$	*Cldn17*$^{-/-}$
n1	2	2.5	3
n2	1	1	3
n3	2	2	3
**Average**	1.666666667	1.833333333	3

C

Bowman's capsule area (average values of 3 fields/mouse)			
	Cldn17$^{+/+}$	*Cldn17*$^{+/-}$	*Cldn17*$^{-/-}$
n1	2.094	3.767667	4.279
n2	1.101667	3.079667	4.082667
n3	1.685667	2.443	3.897
**Average**	1.627111	3.096778	4.086222

Supplementary Figure S3. *Cldn17*$^{-/-}$ mice display kidney injury. **(A)** Hematoxylin and eosin-stained images of kidneys with magnified Bowman's capsule area from WT and *Cldn17*$^{-/-}$ mice. **(B)** Table showing kidney injury scores determined by blinded reviewers (Average of 3 random fields each sample; n=3). **(C)** Table showing showing quantification of Bowman's capsule area by Image J software (Average of 3 random fields each sample; n=3).

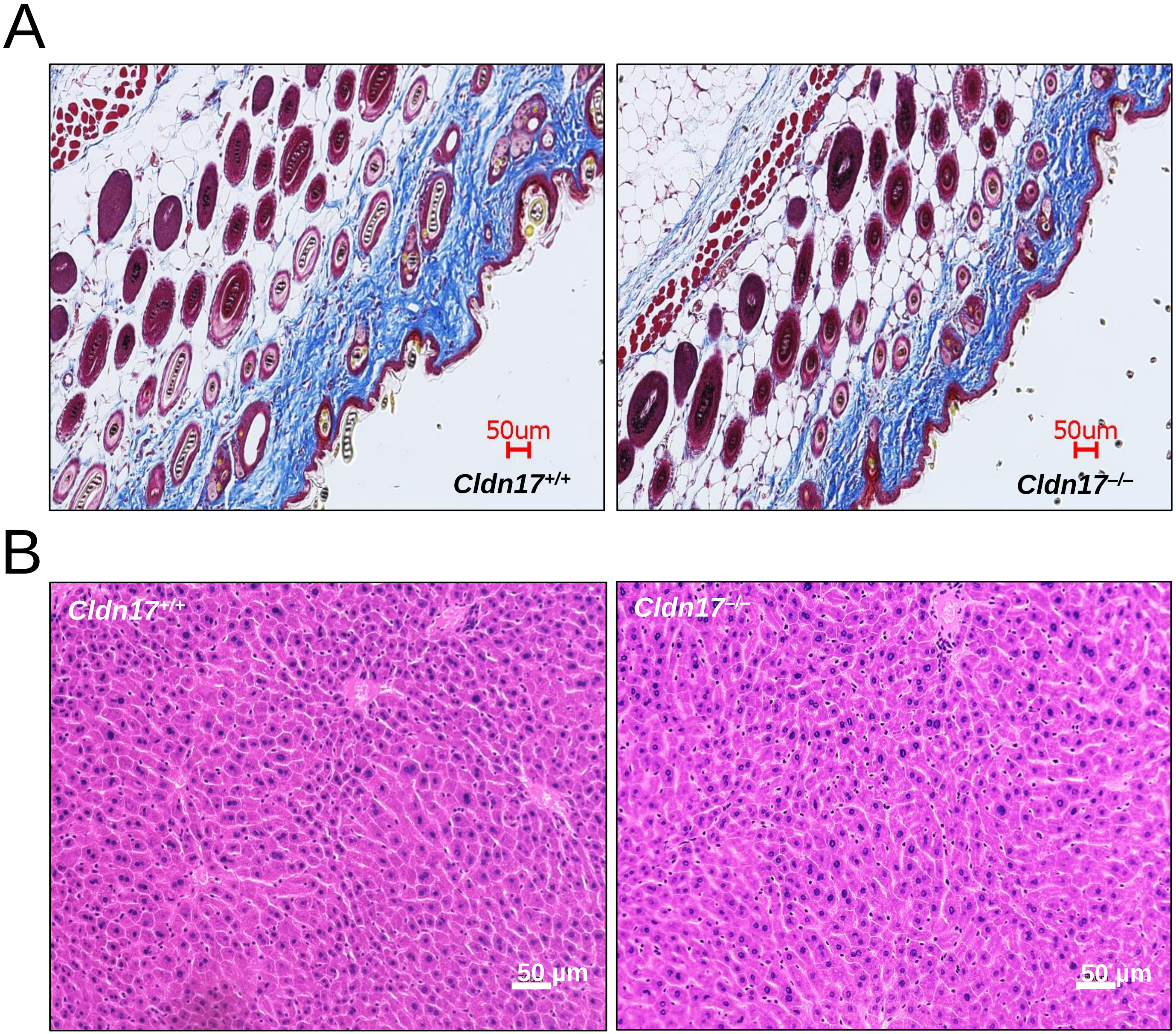

Supplementary Figure S4. Effect of *Cldn17* loss on skin and liver. A. Masson's trichrome-stained skin images. **B.** H&E-stained liver images.

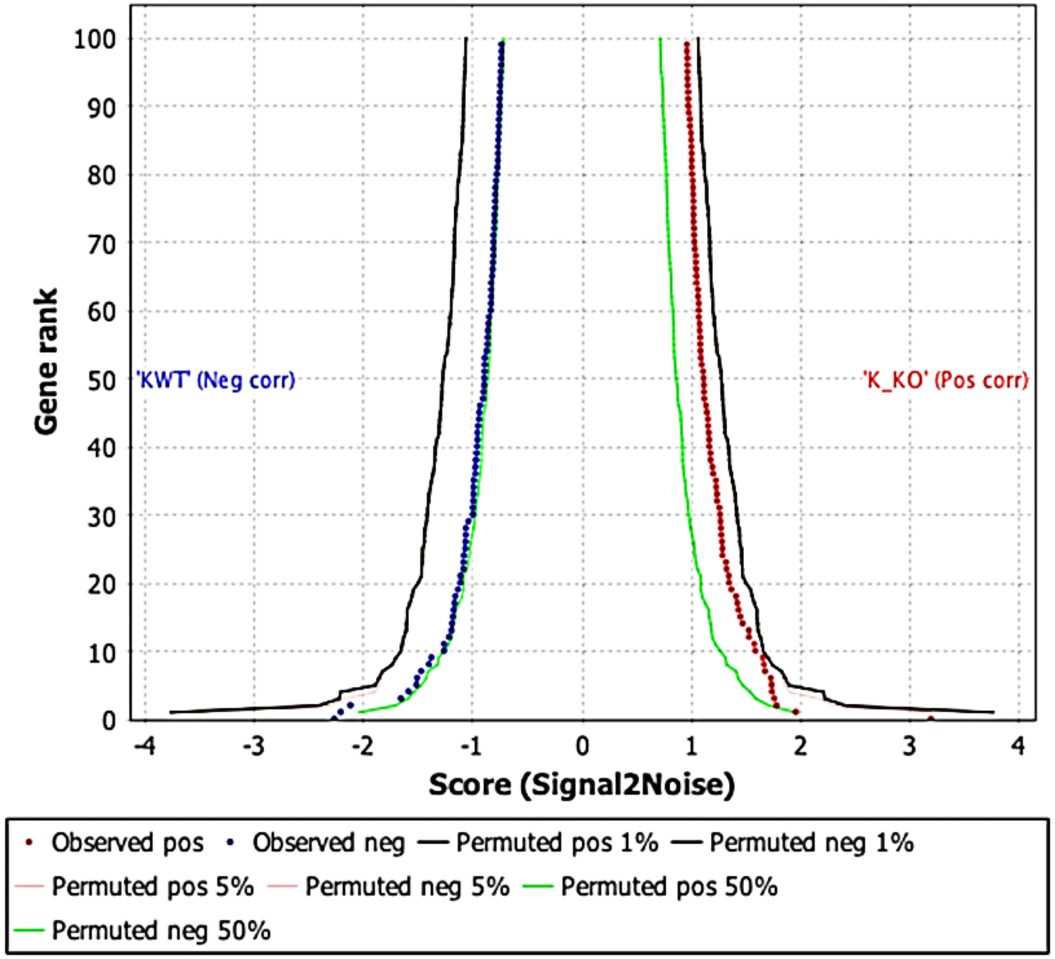

Supplementary Figure S5. The butterfly plot for modulated genes in WTs and Cldn17 knockout groups. Significantly altered genes are represented in red whereas rest of the genes are depicted in green. X-axis signifies fold-changes with upregulation in positive and downregulation in negative values. n=3; $p<0.05$.

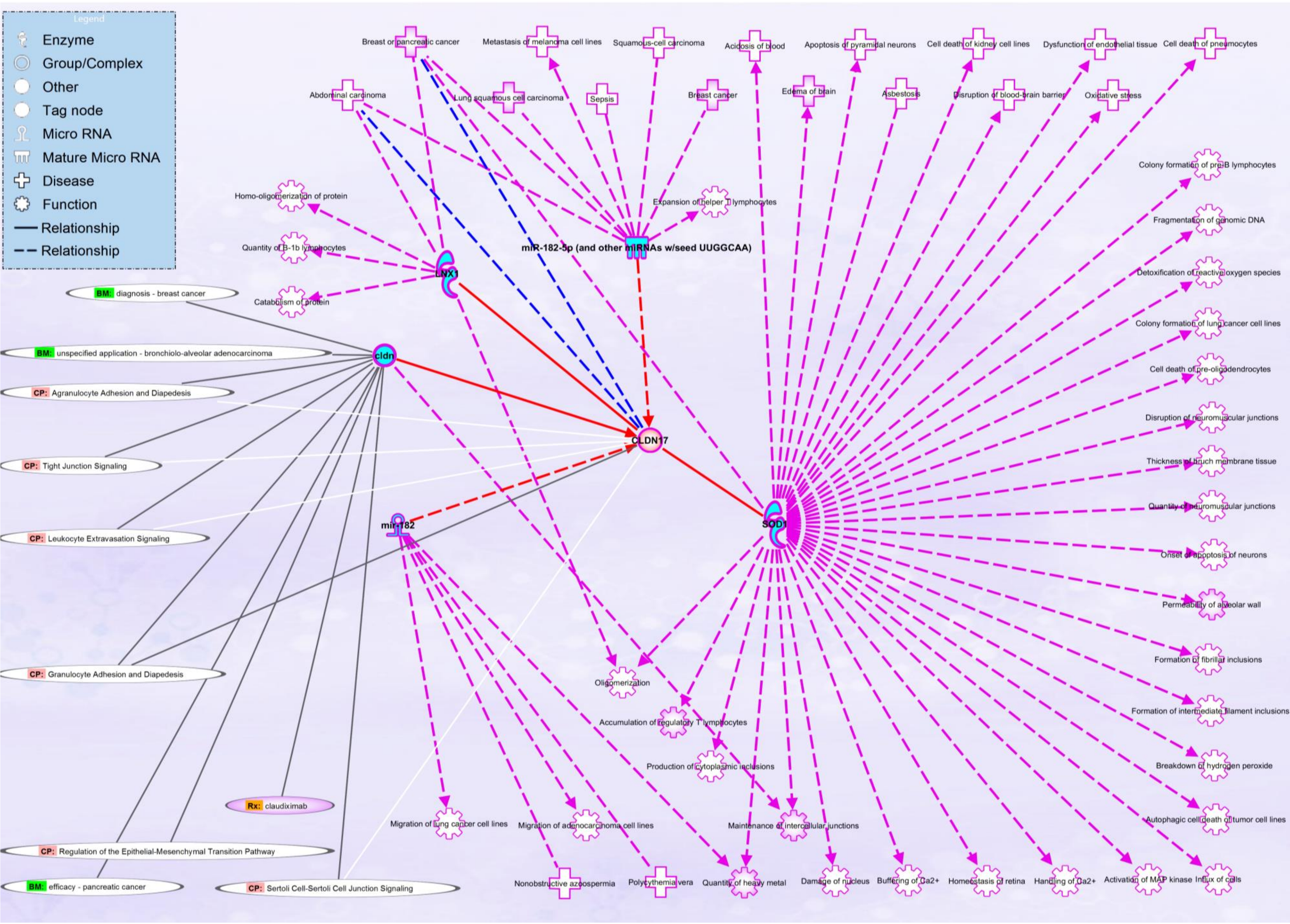

Supplementary Figure S6: Ingenuity pathway analysis predicting a potential link between *CLDN17* and SOD1.

Primer Set	Sequence – 5’ - 3’
Cldn17 Cel1 F	AGAAGACCAGGCACTCCTCT
Cldn17 Cel1 R	TCATGCCGCAGATGCCAATA

↓

Preparation of a PCR reaction with the following reaction components:

Reaction components	Volume (µl)	Final concentrati on
DNA template	1	--
10 µM Primer Cel-1 F	5	1 µM
10 µM Primer Cel-1 R	5	1 µM
Sigma JumpStart™ Taq ReadyMix™ (P2893)	25	1X
ddH_2O	14	--
	50 µl reaction	

↓

PCR cycling conditions:

Cycle Step	Temp (°C)	Time	
1	95	2 min	
2	95	30 sec	x 35 cycles
3	60	30 sec	x 35 cycles
4	68	40 sec	x 35 cycles
5	68	5 min	
6	4	∞	

**PCR products should be treated with Exo-SAP and sent directly to sequencing. SAP will work to dephosphorylate any residual primers and dNTPs (from the PCR) and ExoI will degrade the single stranded molecules so that the DNA can be used directly for sequencing with the specific sequencing primer.

Resolve PCR product on a 1.5% agarose gel.
Expected wildtype band = 408bp

Supplementary Figure S7: Identification of wild-type, heterozygote, and homozygote animals with small deletions (1bp-200bp)

Supplementary Table S1. No changes in the expression of vasopressin genes in the kidneys of *Cldn17*$^{-/-}$ mice (n=3)

Gene Name	*Cldn17*$^{+/+}$	*Cldn17*$^{-/-}$	log2 (fold change)	p-value
**Avpi1**	17.9509	16.201	-0.14798	0.5464
**Avpr2**	5.56332	6.95275	0.321637	0.3123
**Avpr1a**	5.45037	5.70565	0.066038	0.78925

Supplementary Table S2. No changes in the expression of Angiotensin genes in the kidneys of *Cldn17*$^{-/-}$ mice (n=3)

Gene Name	*Cldn17*$^{+/+}$	*Cldn17*$^{-/-}$	log2 (fold change)	p-value
**Agtr1a**	8.30817	7.6259	-0.12362	0.50175
**Agtrap**	20.5049	18.0406	-0.18472	0.14475
**Arrb1**	67.8613	67.1582	-0.01503	0.9055
**Arrb2**	13.9833	16.2582	0.217469	0.1628
**Ace**	259.306	255.746	-0.01994	0.89205

Supplementary Table S3. RT-PCR Primers for mouse Claudins

Gene	Forward Primer sequence (5'- 3')	Reverse Primer sequence (5'- 3')
**Cldn1**	GATGTGGATGGCTGTCATTG	CGTGGTGTTGGGTAAGAGGT
**Cldn2**	ATGGTGACGTCCAGTGCAAT	GGCGAGTAGAAGTCCCGAAG
**Cldn3**	GAGATGGGAGCTGGGTTGTA	GTAGTCCTTGCGGTCGTAGG
**Cldn4**	CCCGAGCCCTTATGGTCATC	GAGTAGGGCTTGTCGTTGCT
**Cldn5**	CTGGACCACAACATCGTGAC	GCCGGTCCAGGTAACAAAGA
**Cldn6**	GAGCCAAGTGCACTACCTGT	TGGTGGGATATTCGGAGGGT
**Cldn7**	GCGACAACATCATCACAGCC	TACCAAGGCAGCAAGACCTG
**Cldn8**	TTGCTGACAGCCGGAATCAT	TCGGAGATCTCTTTTCGGCG
**Cldn9**	CCAAGGCCCGTATCGTACTC	GGACACGTACAGCAGAGGAG
**Cldn10**	GCTAAAATTGCTTGCTTGGC	TCCGTTGTATGTGTAGCCCA
**Cldn11**	TAGCCACTTGCCTTCAGGTG	GAGCAGCAGACGATGACACA
**Cldn12**	AACTGGCCAAGTGTCTGGTC	AGACCCCCTGAGCTAGCAAT
**Cldn13**	CCCTGTCCTGGGTAACACAC	GGCTCCTGAAGGTCTAGCAC
**Cldn14**	GCACAGGCATCTACCAGTGT	TGAGGAGATGAAGCCCAGGT
**Cldn15**	TGTCTACGGTCCATGGCAAC	CCACGAGATAGCCACCATCC
**Cldn16**	CCTGCGATGAGTACGACTCC	CTCCTGCCAAAAAGCAACCC
**Cldn17**	TCTCCCTCCGGTACTGGAAG	GCTCCTCCAAGTTCTCGCTT
**Cldn18**	GTGTCCATCTTCGCCCTGAA	GGTGTACCTGGTCTGAACGG
**Cldn19**	TTCTTCAACCCCAGCACTCC	GCGGGCAACTTAACAACAGG
**Cldn20**	CTTTCATCCTGGCCGTGTCT	GGCTTTCCGGAATGGTCAGA
**β-Actin**	TTTGAGACCTTCAACACCCC	ATAGCTCTTCTCCAGGGAGG
**GAPDH**	CATCACTGCCACCCAGAAGACTG	ATGCCAGTGAGCTTCCCGTTCAG
